# The 'Neglected Tropical Diseases': now a brand identity; responsibilities, context and promise

**DOI:** 10.1186/1756-3305-5-23

**Published:** 2012-01-30

**Authors:** David H Molyneux

**Affiliations:** 1 Centre for Neglected Tropical Diseases, Liverpool School of Tropical Medicine, Pembroke Place, Liverpool, L3 5QA, UK

## Editorial

On 30 January, Bill Gates, Margaret Chan, the Director-General of the World Health Organization, CEOs of major pharmaceutical companies, senior government officials from donor and endemic countries gathered in London together with NGOs and academic institutions, at a meeting entitled **"Uniting to Combat NTDs: Ending the Neglect and Reaching the 2020 Goals" **http://www.unitingtocombatntds.org to announce a massive expansion of the support to control and potentially eliminate or eradicate a group of seventeen Neglected Tropical Diseases. The meeting was convened by the Bill and Melinda Gates Foundation. Since 2004 there has been a gradual but increasing recognition of the importance of neglected tropical diseases (NTDs) as an impediment to development. If the Millennium Development Goals (MDGs), which relate to health, are to be achieved then controlling only HIV, tuberculosis and malaria will not alone address the needs of the poorest. It is essential the so called "other diseases" of the MDGs are given a high priority on the global Health agenda. The London meeting is the culmination of the efforts by many interested partners from diverse constituencies. These groups working together, since the publication in 2010 of the first WHO Report" Working to overcome neglected Tropical Diseases" have recognised the importance of NTDs as impediments to development. It is also important to note that these conditions touch the "bottom billion" but can be addressed either with available tools or through targeted research to develop new products and tools within an acceptable time frame to address well defined needs. Endemic countries, international agencies, bilateral and philanthropic donors, academia and non- governmental organisations as well as existing global partnership organisations are coming together- all committed to achieving unprecedented cooperation to solve a major health problem. The London meeting was a landmark in public health cooperation setting the agenda for the next decade. The commitments made will bring hope providing the final nail in the coffin of diseases which have plagued humanity for millennia. All parties present endorsed the "London" Declaration inspired by WHO's Road Map on NTDs published on the 26^th ^January. The road map sets targets for the period 2012-2020. WHO believes that despite the complexity of the NTDs, the targets are achievable. The Declaration commits partners to sustain and expand resources to achieve ambitious but well- defined and obtainable eradication, elimination or control targets. Partners will advance research and development, enhance collaboration on NTDs at national and international levels, work more effectively together, enable adequate funding for endemic countries to implement programmes, provide the technical support for evaluation and monitoring and provide regular updates towards the 2020 goals. The diseases embraced by this global movement do not have any formally structured single partnership but all constituencies share a strong commitment to support WHO's strategies, goals and targets.

These diseases have not suddenly emerged; they have been the subject of studies since the earliest days of the discipline of Tropical Medicine where the father of that discipline, Sir Patrick Manson, first discovered that insects were capable of transmitting infective agents when he discovered the mosquito transmission of filarial infections in China in 1879. During the last 8 years the messages articulated by advocates of NTD eradication, elimination and control have gradually been recognised by global health leaders and policy makers as amongst the best investments that a health dollar can buy. The term Neglected Diseases emerged in the 1980's when the late Dr Ken Warren as Head of the Rockefeller health department introduced the term Great Neglected Diseases. The term fell into abeyance until it was adopted at the Berlin meetings of 2003 and 2004 organised by WHO and Deutsche Gesellschaft fur Technissche (now Internationale) Zusammenarbeit (GTZ). WHO then created a specific Department of Control of Neglected Tropical Diseases and started to develop policies recognising the need for integration of approaches and the increasing volume and diversity of drug donations, culminating in the huge increase in the donations. The transition from neglect to a high level of priority can be attributed to a concerted effort on the part of partners who have embraced the message of need, burden of disease, availability of effective donated and safe drugs and the proven successes. Following the meeting in London substantial progress to address the needs for NTD eradication, elimination and control have been met. However, partnership, innovation, and additional resources will still be needed. This is a great leap forward while still keeping the space for more to join a transformative movement by "working together in an innovative, flexible and cost effective way" as articulated by the Margaret Chan, the Director General of WHO. New commitments announced in London of particular importance are the expanded donations of praziquantel for schistosomiasis, the most important tropical disease in Africa after malaria, and ambisome for visceral leishmaniasis. In the broader context that twelve of the worlds biggest pharmaceutical companies have collectively committed to extend their donations through 2020 to help meet the WHO control and elimination goals is remarkable. Earlier in January the UK Department for International Development announced a five- fold increase in resources for NTDs. The resources for Guinea Worm Eradication have been fully provided for by a consortium of donors-the UK Department for International Development, the Bill and Melinda Gates Foundation, the President of the United Arab Emirates and the Children's Investment Fund Foundation. In addition the World Bank will offer NTD endemic countries new resources to build community health systems as well as work to expand the Onchocerciasis Trust Fund to other NTDs.

Two years ago I wrote a Comment for the Lancet introducing a Series on NTDs entitled "Neglected Tropical Diseases-beyond the tipping point?" Now I believe it would be safe to remove that question mark. The London meeting with its remarkable commitments from all partners and the accompanying Declaration is a Global Health turning point. A decade ago few programmes had adequate resources for implementation and were acting in isolation. There was limited cross- talk between the disease specific communities or recognition of the synergistic impacts of drugs, the tools for evaluation were less refined, research was poorly funded and less focussed, there was limited NGDO involvement and coordination and less endemic country commitment- there has been a "sea change". NGDOs have also been "visionary"; those which a traditionally focussed on blindness have recognised that the systems in place for implementation of ivermectin for onchocerciasis control can also address other disabilities.

A global consensus that actually addresses the low hanging fruit of NTD control, elimination and eradication with the largest donations ever made by a group of all the major pharmaceutical donors is a vital contribution to health and development. This is real and transparent progress made over a decade when the existing programmes were truly neglected and struggling for recognition on the Global Health agenda. The change of paradigm to the concept of an "NTD" brand based on integrated control or elimination was a crucial decision. This was communicated through strong and visionary leadership in the World Health Organization (WHO) who established an NTD Department. This was a critical bedrock for consistent policy and leadership as new policies and norms were articulated- a pre-requisite to advocate for the necessary donations. It is important to recall as we move towards a new era of elimination of at least some NTDs that the principles and rationale for pro-poor and non-discriminatory interventions also relate to equity, human rights, access to essential medicines, strengthening of health systems and enhanced capacity development.

Over recent years there has been massive up-scaling of country commitment, proven successes of implementation strategies, increased drug donations and hugely increased bilateral and philanthropic support. The London Declaration provides the platform for the next 8 years- a statement with which no one can argue. However, whatever the perceived success of reaching the level of recognition that the NTD brand has now achieved we must look forward. As a committed community we have now acquired new resources and brand identity. This brings responsibility and obligations.

A primary responsibility is to our donors, the taxpayers of the developed world struggling with economic uncertainty. It is remarkable that there has been such a generous responses from the UK and US governments in the current environment. It is essential that we provide value for money; the taxpayer identifies with HIV/AIDS, malaria and TB but certainly less with the NTDs. Our argument for taking that responsibility is that we provide more health to more people for less money. As a community it behoves us to remember that the value of the donated products from the pharmaceutical partners is between US$2-3 BILLION annually. Another responsibility is to ensure that these donations are used efficiently and effectively and this links to the final responsibility- of ensuring the drugs reach the communities who are the ultimate beneficiaries; NTD programmes based on preventive chemotherapy have an enviable therapeutic coverage record but we know we have to go further and faster by expanding geographic coverage.

These responsibilities should be driving our thinking. What should we have now on the agenda as we look towards the 2020 horizon? Clearly we need to maintain our advocacy and build on successes. We need to do several things to maintain the momentum and fulfil our responsibilities.

**First**, we must manage these huge drug donations in the most effective way. These precious, high quality commodities for the most neglected populations are the key to our progress and success and are a major reason for the position we now find ourselves in. Poor people, as Roy Vagelos knew when he announced the donation of ivermectin in 1987, cannot afford even the cheapest of drugs; the only way to satisfy demand was to donate them. That decision still deserves recognition as it was the starting point for the current movement. In the coming years WHO has a key role to play maintaining its core leadership function of developing policy and responding to and representing member states and ensuring the drug donations are effectively managed. This is critical.

**Secondly**, we must, if we are to succeed, find a new way of working together to reflect the new reality of financial resources; this will require flexibility, open and transparent communication and above all the need to subjugate our institutional and organisational sensitivities to achieve the greater good. New models and thinking will be required. This will be one of the guiding principles of the newly established UK Coalition against NTDs which brings together UK strengths from the different interested sectors http://www.ntd-coalition.org. Academia, NGDOs, the education sector and the different disease specific communities will facilitate cross talk and maximise the leverage we can obtain through their networks. The Coalition pledges to promote the London Declaration by words and actions and work with all partners, particularly the endemic countries, the pharmaceutical sector and WHO. We believe that the power of the UK Coalition lies in its historic institutional research commitment dating to the early part of the last century together with the NGDO implementation record in partner countries and its traditional association with WHO. In short the impact of the whole will be greater than the constituent parts.

**Thirdly**, a further need we must address is the deficit in human capacity. This is not just programme management capacity but the NTD research capacity in Africa. The European Union Foundation Initiative for NTDs(EFINTD) meeting in Maputo brings together the resources of five Foundations- Cariplo, Gulbenkian, Merieux, Nuffield and Volkswagen, in a unique partnership to provide resources over the longer term for African Fellowships. The current Fellows appointed three years ago have met regularly; they have established there own networks of collaboration, grown in confidence and achieved scientific independence. The Maputo will meeting bring together existing post-doctoral Fellows and will select another group, through a competitive process. Some 20 Fellows are already in place mentored by European institutional partners. This initiative fills a gap and has demonstrated there exists an untapped yet enthusiastic pool of human resources who, given a small amount of support, will be the next generation of scientists to take forward both the basic and operational research agendas for NTDs and articulate future policy.

**Fourthly**, the NTD community cannot stand still. We have disease specific targets and the constituencies who are fully aware of the research needs; those communities are profoundly grateful for the support from the Bill and Melinda Gates Foundation as well as other Foundations and funding agencies which support basic and applied research. The needs are well recognised. Complacency about the efficacy of existing products is not appropriate as there is rarely a perfect tool. Given the long time- lines of getting new products into practice we have the obligation to move quickly with the products we have and to be aware of the potential risks and pitfalls. These can be tackled, as has been often demonstrated. However, there are other areas of neglect that we need to address. We have forgotten the neglected zoonotic diseases which interface with the many millions of those dependent on livestock as their sole asset and whose livestock, as well as themselves, succumb to zoonotic infections which are often undiagnosed and unreported. There is also the added dimension of co-morbidity of NTDs. In particular that of the burden of mental illness imposed on the sufferers themselves and their carers. The numbers and consequent burden of depression engendered by the stigma, social exclusion and economic impact has not been considered or addressed. A further dimension is the need for health systems research a difficult subject to address and often setting specific and hence difficult for results to be generalisable. These are just some ideas for consideration as we move forward towards 2020.

In conclusion I must focus on the most important issue - that of equity so elegantly expressed in the quote on the front cover of the Lancet two years ago following Bernhard Liese's work **"Only 0.6% of overseas development assistance for health is allocated to neglected tropical diseases, despite such diseases affecting at least 1 billion people"**. This figure is and remains unacceptable when, for such trivial sums, we can make a massive impact. The recent commitments may indeed go some way to changing the 0.6% figure. However, in terms of the donated drugs which have been such an important a base for the NTD momentum I come back to the view that if we, as an international community of partners, cannot deliver high quality, donated drugs which have proven multiple impacts then we cannot expect more complex problems of international health to be solved. The NTD community has the opportunity over the next few years to make the massive progress towards eliminating permanently diseases which for far too long have been more than neglected, they have been ignored.

## Author information

Professor David Molyneux,

Centre for Neglected Tropical Diseases,

Liverpool School of Tropical Medicine.

Interim Chair, UK Coalition against Neglected Tropical Diseases

